# Clinical exome sequencing identifies two novel mutations of the *SCN1A* and *SCN2A* genes in Moroccan patients with epilepsy: a case series

**DOI:** 10.1186/s13256-019-2203-8

**Published:** 2019-08-23

**Authors:** Maryem Sahli, Abdelali Zrhidri, Siham Chafai Elaloui, Wiam Smaili, Jaber Lyahyai, Fatima Zohra Oudghiri, Abdelaziz Sefiani

**Affiliations:** 1 Centre de Recherche en Génomique des Pathologies Humaines (GENOPATH), Faculté de Médecine et de Pharmacie, Mohammed V University of Rabat, 10100 Rabat, Morocco; 2 Département de génétique médicale, Institut National d’Hygiène, BP 769 Agdal, 10090 Rabat, Morocco; 3 Unité de Neuropédiatrie, Hôpital d’Enfants de Rabat, Rabat, Morocco; 4 Département de Génétique Médicale, Institut National d’Hygiène, 7Avenue Ibn Batouta, B.P. 769, 11400 Rabat, Morocco

**Keywords:** Epilepsy, Clinical exome sequencing, *SCN1A*, *SCN2A*, Novel mutation, Moroccan

## Abstract

**Background:**

Epilepsy is the most common neurological disorder that causes spontaneous, unprovoked, and recurrent seizures. Epilepsy is clinically and genetically heterogeneous with various modes of inheritance. The complexity of epilepsy presents a challenge and identification of the causal genetic mutation allows diagnosis, genetic counseling, predicting prognosis, and, in some cases, treatment decisions. Clinical exome sequencing is actually becoming a powerful approach for molecular diagnosis of heterogeneous neurological disorders in clinical practice.

**Case presentation:**

We report our observations of three unrelated Moroccan patients referred to our genetics department for molecular diagnosis of epilepsy: a 4-year-old Moroccan boy, a 3-year-old Moroccan girl, and a 7-year-old Moroccan boy. Due to the heterogeneity and complexity of epilepsy, we performed clinical exome sequencing followed by targeted analysis of 936 epilepsy genes. A total of three mutations were identified in known epilepsy genes (*SCN1A, SCN2A*). By clinical exome sequencing, we identified two novel mutations: c.4973C>A (p.Thr1658Lys) in *SCN1A* gene and c.1283A>G (p.Tyr428Cys) in the *SCN2A* gene, whereas the third mutation c.3295G>T (p.Glu1099*) was already described in patients with Dravet syndrome.

**Conclusion:**

This study demonstrates that clinical exome sequencing is an effective diagnosis tool to investigate this group of diseases with huge diversity and defends its use in clinical routine.

## Background

Epilepsy or “seizures disorders” is the most common neurological disorder. The prevalence of epilepsy is 6.38 per 1000 individuals, and approximately 50 million people are affected worldwide [1, 2]. Epilepsy includes a large neurological condition characterized by spontaneous, unprovoked, recurrent, and unpredictable seizures [3]. Epilepsy can be isolated or a clinical feature of a syndrome with additional signs [4].

The causes of epilepsy are multiple. The causes involve genetic factors in 70–80% of cases of epilepsy, which constitute the main factor in idiopathic epilepsies [5]. On the other hand, a large proportion of cases of epilepsy are caused by acquired conditions such as cerebral trauma, cerebral tumor, cerebral infection, cerebrovascular disorders, and inborn errors of metabolism [6]. Because of the phenotypic and genetic heterogeneity of this group of diseases, we opted for clinical exome sequencing (CES) as a diagnosis approach to allow a genetic diagnosis for our patients.

We report here the observations of three unrelated patients referred to our Department of Medical Genetics for epilepsy of undetermined origin, for genetic study. As a result, we identified causal mutations in the three analyzed patients, including two novel mutations.

## Case presentation

### Patients

Three patients were referred to our Department of Medical Genetics in Rabat for epilepsy without previous clinical diagnosis. Informed parental consent for genetic testing was obtained for all participants of this study. Clinical diagnoses and all detected variants are summarized in Table 1.

**Table 1:** Clinical and genetic findings of the three Moroccan patients with epilepsy

Number	Patient 1 (P1)	Patient 2 (P2)	Patient 3 (P3)
Age	4 years	3 years	7 years
Age at onset	6 months	5 months	21 months
Sex	M	F	M
Consanguineous	No	No	No
Psychomotor development retardation	Yes	Yes	Yes
Interictal EEG	Extended EEG isolated points are seen over the frontocentral region	NA	Tonic seizures
Cerebral MRI	N	N	N
Drugs resistance	Yes	Yes	No
Gene/Transcript	*SCN1A*/NM_001202435	*SCN1A*/NM_001202435	*SCN2A/*NM_021007.2
Nucleotide change	c.4973C>A	c.3295G>T	c.1283A>G
Amino acid change	p.Thr1658Lys	p.Glu1099*	p.Tyr428Cys
Inheritance	AD	AD	AD
Origin of variant	*De novo*	*De novo*	*De novo*

*AD* autosomal dominant, *EEG* electroencephalogram, *F* female, *M* male, *MRI* magnetic resonance imaging, *N* normal, *NA* not available

### Patient 1

Our first patient is a 4-year-old Moroccan boy, non-consanguineous, the last child of three siblings, referred for Dravet syndrome. His history showed normal psychomotor development until 6 months of age. Daily generalized seizures started at 6 months of life and were treated with sodium valproate, clobazam, and carbamazepine but without any response. At clinical examination, growth parameters were within normal limits with no facial dysmorphia. Magnetic resonance imaging (MRI) was normal; however, an electroencephalogram (EEG) showed an extended EEG and isolated points were seen over the frontocentral region.

### Patient 2

Our second patient is a 3-year-old Moroccan girl referred to our Department of Medical Genetics for uncontrolled seizures. She was born at term after an uneventful pregnancy; her birth weight was 3.2 kg. She was the only child of a non-consanguineous couple. At the age of 5 months, she developed generalized seizure following a fever. She was treated with sodium valproate and clobazam but without any response. At clinical examination, she had stature and weight delay at < third percentile, microcephaly at − 2SD, with no facial dysmorphia. MRI scans showed normal results.

### Patient 3

Our third patient is a 7-year-old Moroccan boy who presented to our department with idiopathic epilepsy and developmental regression. His history showed normal psychomotor development until 21 months of life. Treatment with sodium valproate and clobazam was initiated with poor seizure control. At physical examination, he had stature at < third percentile, microcephaly at − 2SD, with no facial dysmorphia. An MRI was normal but EEG showed generalized tonic seizures.

### CES and data analysis

We tested three unrelated patients with epilepsy of undetermined origin. Due to the phenotypic and genetic heterogeneity of epilepsy and no hot spots mutation, we used CES as a diagnosis strategy for individuals with this clinical entity.

The CES was performed using the SOPHiA™ Genetics CESV1 kit on NextSeq 500 (Illumina). Raw data were analyzed, annotated, and filtering steps were performed using SOPHiA™ DDM (SOPHiA Genetics®). Variants with allele frequencies above 2% in Exome Sequencing Project (ESP) 6500, and variants not predicted to be deleterious were excluded. CES results were further validated using Sanger sequencing by designing the primers of the identified mutations. Amplification products were electrophoresed on 1% agarose gels. Sanger sequencing was done with dye terminator chemistry (ABI Prism® BigDye v3.1) and run on automated sequencer Applied Biosystems Prism 3130 DNA Analyzer. Obtained sequences were aligned to the reference genome (GRCh37/hg19) using DNA Variant analysis software (Mutation Surveyor® software).

The established variants were cross-checked with the 1000 Genomes Project database (http://www.1000genomes.org/), with the Exome Variant Server (http://evs.gs.washington.edu/EVS/), Human Gene Mutation Database **(**HGMD; http://www.biobase-international.com/product/hgmd), and with the “ClinVar” database (http://www.ncbi.nlm.nih.gov/clinvar/).

## Discussion

Epilepsy is the most common neurological disorder; it is characterized by a broad clinical and genetic spectrum. The clinical heterogeneity and molecular complexity of this group of diseases pose a real challenge to clinicians for diagnosis and treatment. CES is actually becoming a powerful approach for molecular diagnosis of heterogeneous neurological disorders such as epilepsy in clinical practice. Therefore, it is considered a new approach of molecular diagnosis to identify mutations in patients with rare and heterogeneous genetic diseases facing a large genetic differential diagnosis [7].

Due to the phenotypic and genetic heterogeneity of neurological disorders, especially epilepsy, many teams use gene panels as a diagnosis approach. However, given the large number of genetic diseases with epilepsy as a symptom and given that gene panels developed by each laboratory differ greatly, CES is becoming an effective common molecular diagnostic tool for individuals with a heterogeneous phenotype. In addition, epilepsy gene panels are multiple and so far there are no next-generation sequencing (NGS) guidelines for patients with epilepsy [8]. Currently, there is a huge diversity in the number and composition of genes of the different epilepsy panels commercially available [9–12]. Gene panels are also expensive and associated with a low diagnosis yield [13].

CES analysis is associated with a decrease in cost and a high diagnosis yield in comparison to other genetic tests used currently in diagnosis, including sequencing the whole gene and gene panels, and could be used in patients with epilepsy to identify mutations and allow a definitive diagnosis, to adapt antiepileptic drug, to predict the prognosis, and to provide genetic counseling to families.

As a department of medical genetics providing genetic services and testing for various diseases in a country with lower income, molecular diagnosis strategies by NGS or single gene sequencing with Sanger of heterogeneous diseases like epilepsy may represent a real challenge. It is obvious that we design an appropriate panel of each disease and that would be highly efficient. However, the very limited number of cases of epilepsy among the various genetic diseases from our consultation and the significant cost of dedicated panels bring us to consider a customized multigenes panel approach. This strategy of diagnosis with CES is better adapted to our diversified requests of molecular tests.

In this study, we performed CES in three unrelated patients with epilepsy of undetermined origin. We detected causal mutations in all cases; the three mutations were found in two genes, *SCN1A* and *SCN2A*, which encode the brain sodium channel. Two mutations were novel findings, highlighting the value of CES to detect *de novo* mutations especially when applied to heterogeneous disorders such as epilepsy.

In our first patient, a heterozygous mutation was identified: c.4973C>A (p.Thr1658Lys) of the *SCN1A* gene (NM_001202435) (see Table 1). This variant has never been reported in public databases; however, the change of amino acid threonine in position 1658 to another amino acid has been reported several times in patients with Dravet syndrome. The p.Thr1658Lys of the *SCN1A* gene was predicted to be probably damaging by a bioinformatic analysis using PolyPhen and SIFT tools. PolyPhen predicted it as possibly damaging, while SIFT predicted it as deleterious or disease causing.

Our second patient had a heterozygous mutation c.3295G>T (p.Glu1099*) of the *SCN1A* gene (see Table 1), this mutation was already identified in patients showing Dravet syndrome [14].

For our third patient we detected a single heterozygous mutation in coding exon 10: c.1283A>G (p.Tyr428Cys) of the *SCN2A* gene (NM_021007.2) (see Table 1). The mutation in the *SCN2A* gene in our patient is considered pathogenic because it is a *de novo* mutation and occurred in an evolutionary conserved amino acid at this position of the protein. In addition, this variant was absent from the control population (Exome Aggregation Consortium, Cambridge, MA). The c.1283A>G of the *SCN2A* gene is a novel mutation, and it had never been reported in the literature to the best of our knowledge.

## Conclusion

In conclusion, these observations showed that CES is a powerful tool to detect *de novo* mutations, especially when applied to heterogeneous neurologic phenotypes, such as epilepsy, facing a broad genetic differential diagnosis and defend its application in clinical practice.

## Data Availability

Available on request.

## Abbreviations

CES: Clinical exome sequencing
EEG: Electroencephalogram
MRI: Magnetic resonance imaging
NGS: Next-generation sequencing
